# Effects of behavioural activation on substance use and depression: a systematic review

**DOI:** 10.1186/s13011-018-0173-2

**Published:** 2018-09-29

**Authors:** Carmela Martínez-Vispo, Úrsula Martínez, Ana López-Durán, Elena Fernández del Río, Elisardo Becoña

**Affiliations:** 1 Smoking Cessation and Addictive Disorders Unit, Department of Clinical Psychology and Psychobiology, Faculty of Psychology, University of Santiago de Compostela, Santiago de Compostela, Spain; 2 Tobacco Research and Intervention Program. Department of Health Outcomes and Behaviour, H. Lee Moffitt Cancer Center, Fl, Tampa, USA; 3 Department of Psychology and Sociology, University of Zaragoza, Zaragoza, Spain

**Keywords:** Behaviour therapy, Behavioural activation, Substance use disorder, Depression, Systematic review

## Abstract

**Introduction:**

Substance use and depression co-occurrence is a frequent phenomenon and an important public health concern. Given the clinical implications and the high prevalence of both disorders, effective interventions are needed.

**Methods:**

The aim of this study is to review Behavioural Activation (BA) intervention effects to improve substance use behaviour and depression. A systematic review was conducted using MEDLINE, EMBASE, and PsycINFO. The Effective Public Health Practice Project Quality Assessment Tool (EPHPP) was used to assess the methodological quality of included studies. Two authors independently screened titles and abstracts, reviewed selected studies, and extracted data.

**Results:**

Of the 7286 studies identified, eight met inclusion criteria. Designs of the studies included six randomized controlled trials (RCTs), and two pre-post design studies. One trial received weak methodological quality, six moderate, and one strong. Three studies addressed smoking behaviour; two targeted opiate dependence; two focused on alcohol/drug dependence; and, one on crystal methamphetamine abuse. Results showed that BA had a positive effect on substance use outcomes in seven of the eight reviewed studies, and improved depression over time in six studies.

**Conclusions:**

Although studies conducted so far are limited by their heterogeneity and sample sizes, results are promising. There is a need of well controlled and powered studies to establish and to confirm the effectiveness of BA for the treatment of substance use and depression. Future studies should include stronger methodological designs, larger sample sizes, and long-term follow-ups.

**Trial registration:**

PROSPERO registration number: CRD42016039412.

**Electronic supplementary material:**

The online version of this article (10.1186/s13011-018-0173-2) contains supplementary material, which is available to authorized users.

## Introduction

Substance use disorders (SUDs) and mental health disorders are significant contributors to the global burden of disease, and their impact is increasing over the years both in high-income and low-to-middle-income countries [1]. In fact, prevalence of SUDs reaches the 8.7% of U.S. adults, and among the most prevalent mental disorders stand out depression, with about 6.7% of U.S. adults having a major depressive episode during the past year [2].

In the general population, co-occurrence of SUDs (including alcohol, tobacco, cannabis, cocaine, and other illicit drugs) and depression is a common and well documented phenomenon [2–6]. In this sense, a meta-analysis of epidemiological studies of the comorbidity of SUDs and mood and anxiety disorders [7], found a strong association between major depression and several SUDs such as alcohol use disorders (pooled Odd Ratio [OR] = 2.42) and illicit drug use disorder (pooled OR = 3.80). Similarly, research has shown that people with SUDs is more likely to have a major depressive disorder, compare to those without SUDs, even after controlling for sociodemographic characteristics and additional psychiatric comorbidity (adjusted OR = 1.2 and 1.3, respectively) [8]. Moreover, depression has also been found consistently higher in smokers compared to never smokers (OR = 1.50) and former smokers (OR = 1.76) [9].

Regarding people seeking substance use treatment, depression is a particular concern because of its high prevalence and clinical implications [10–12]. Specifically, depression has been related to greater physical, psychological, and social impairments, poorer treatment adherence, and worse treatment outcomes [13, 14]. Importantly, several studies have found that depression decreases the likelihood of abstinence in people undergoing substance use treatment [15–17].

Previous literature has suggested the existence of common features between substance use disorders and depression. For example, studies have highlighted the key role of positive reinforcement in both conditions [18, 19]. Positive reinforcement can be defined as the process by which a response is followed by a stimulus, and response probability increases. Positive reinforcers are fundamental in this process, and they can be defined as incentives, stimulus, and/or activities that are preferred for an individual. From a behavioural perspective, depression occurs when positive reinforcement for healthy behaviours decreases, there is a low availability of positive reinforcers in the environment, and/or when there is a lack of behavioural skills to achieve them [20]. In the case of SUDs, studies have found that people with SUDs are less engaged in non-drug-related activities and have less alternative positive reinforcers in their environment (e.g., social activities) [18, 21–23]. Indeed, previous research have demonstrated that engaging in alternative activities, as exercise or creative activities, was associated with reductions in substance use consumption [24, 25]. In this line, following the approach of behavioural economic interventions, Murphy et al. [26] found that adding a component addressing substance-free activities (academic, career-related, and leisure activities) to an alcohol brief motivational interviewing session for heavy drinking among college students, was associated with reductions in alcohol problems. Similarly, Reynolds et al. [27] integrated a BA approach within a standard college orientation program and found a significant reduction in the consequences associated with alcohol drinking (e.g., alcohol-related injuries, social and psychological problems).

Due to the high comorbidity between substance use and depression, and the impact of depression on substance use treatment outcomes, it is important to address both disorders simultaneously. There exist different treatments for depression and SUDs, as cognitive behavioural therapy (CBT) or contingence management interventions (CM) [28, 29]. Although some preliminary findings indicate that these interventions have certain efficacy in treating both conditions, there is a need to continue developing and testing interventions for both disorders [30, 31].

Behavioural Activation (BA), which was originally conceptualized as a treatment for depression, is emerging as an option for SUDs. This intervention has its roots in the traditional behaviourism approach, but a renewed interest appeared since the study conducted by Jacobson et al. [32]. In such study, the authors isolated the BA component of cognitive therapy (CT) to determine whether BA by itself could be as effective as CT for depression treatment. Their results confirmed the equal effectiveness of BA and CT in reducing depressive symptoms, pointing out that BA is more parsimonious and less complex than CT. Nowadays, BA is considered a well-established and cost-effective intervention for depression [33, 34].

BA characteristics, and the focus on providing rewarding experiences in daily life different from substance use, make of this approach a potential intervention to increase substance use abstinence outcomes and to relapse prevention [35]. Since BA effectiveness has been widely demonstrated in depression treatment, we sought to extend previous findings by analyzing whether BA would improve comorbid substance use outcomes as well. Therefore, the aim of this review was to analyze the results of BA intervention on (i) substance use, abstinence, or relapse; and on (ii) depression symptom outcomes in individuals with substance use and depression.

## Method

### Search strategy

This systematic review followed the Preferred Reporting Items for Systematic Reviews and Meta-Analysis (PRISMA) statement [36], and the review protocol was registered with PROSPERO (CRD42016039412). The PRISMA checklist is provided in Additional file 1. The following electronic databases were used for the literature search, with alterations to the search strategy for specific databases: MEDLINE, PsycINFO, and Excerpta Medica DataBase (EMBASE). The literature search strategy for the three electronic databases, including any search limits used, is provided in Additional file 2. A search of reference lists of included studies and Google Scholar (first 200 citations published online between January 2000 and May 2018) was undertaken. We included studies published in English and Spanish, and all years available in the selected databases (up to May 2018).

### Study selection criteria

#### Study characteristics

The following study designs were included: (i) experimental studies (randomized controlled trials, quasi-randomized trials, controlled clinical trials); (ii) quasi-experimental studies (interrupted time series, before-and-after studies) and; (iii) observational studies (cohort studies and case-control studies). We excluded case series studies, research protocols, review articles, and non-interventional studies.

#### Participants

Participants of included studies were: (i) adult substance users (age ≥ 18 years); (ii) with depression. For the purpose of this review, substance users were defined as individuals who used substances assessed by a screening questionnaire (e.g., Substance Use Weekly Inventory) or as individuals who met criteria for SUD by a diagnostic interview (e.g., Structured Clinical Interview for Diagnostic and Statistical Manual of Mental Disorders; SCID-DSM). Substances included alcohol, tobacco, caffeine, cannabis, cocaine, heroin, amphetamines, ecstasy, synthetic drugs, and non-prescription use of legal drugs (e.g., morphine, codeine, benzodiazepines). People with depression were defined as individuals who experienced a depressive disorder assessed by a structured clinical interview conducted to internationally recognized standards (e.g., DSM) or depressive symptoms established by a validated screening measure (e.g., Beck Depression Inventory).

#### Type of intervention

Included studies were those examining the effect of face-to-face BA intervention on substance use and depression outcomes. To define BA features, Kanter et al. [37] reviewed the specific treatment components of BA and identified the following: activity monitoring, assessment of life goals and values, activity scheduling, skills training, relaxation training, contingency management, procedures targeting verbal behaviour, and procedures targeting avoidance. Despite the broad range of techniques used in BA interventions, they found that activity monitoring and scheduling were constant components across interventions.

Although there exist several conceptualizations of BA [38, 39], they all focus on behaviour change through the increase of positive reinforcement using strategies to encourage individuals to engage in adaptive and rewarding activities [40]. Therefore, in this review, we used the term ‘BA’ to cover all BA conceptualizations, including those studies where at least activity self-monitoring and scheduling were core elements of the intervention [37].

#### Exclusion criteria

Excluded studies were those in which: (i) participants had cognitive impairment; (ii) intervention was computerized or Internet-delivered; (iii) intervention did not include self-monitoring or activity scheduling; (iv) BA was only one component of CBT and not the core treatment element; and (v) both substance use and depression outcomes were not included.

### Outcomes

Primary outcomes were: (i) substance use (at least one outcome related to substance use, abstinence, or relapse); and (ii) depressive symptoms.

Secondary outcomes were: (i) treatment adherence and retention; and (ii) number of quit attempts, use of other substances, motivation to quit, health-related conditions (e.g., diabetes, Human Immunodeficiency Virus, HIV), other mental health symptoms, and healthcare use. These variables were included as secondary outcomes since they have demonstrated to be predictors of treatment outcomes [41].

### Study selection

**Fig. 1:**
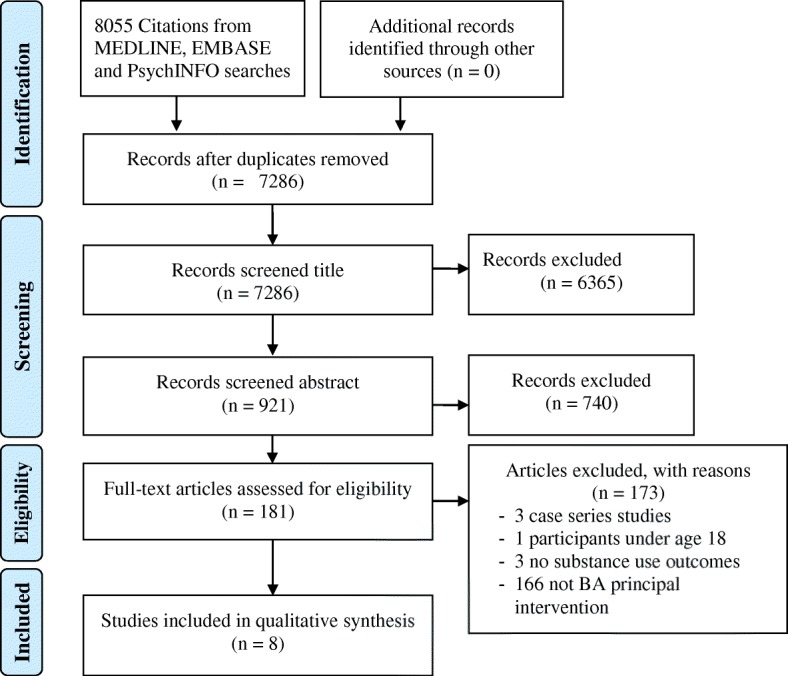
PRISMA flowchart depicting the process of searching, selecting and screening studies according to eligibility criteria

Titles and abstracts retrieved by electronic searches were exported to reference management software (RefWorks) to remove duplicates. References were then exported to the online software tool Covidence for screening. Titles and abstracts were screened independently by two authors (CMV and UM). Disagreements were discussed by the two reviewers. The two reviewers (CMV and UM) performed independently full-text screening, data extraction, and quality assessment. Reasons for full text exclusion were recorded and documented in a PRISMA flow diagram (Fig. 1).

### Data extraction and analysis

Data were extracted independently by CMV and UM using a data extraction form constructed in Microsoft Excel 2010®: study identification features, study design, participant characteristics, sample size, intervention delivery mode, who delivered the intervention, whether the intervention was individual or group sessions, group size for group-based intervention, duration of intervention, number of sessions, length of sessions, treatment setting, depression outcomes, substance use outcomes, and, if reported, information on the components of the BA intervention. Discrepancies were resolved by discussion between the two reviewers.

### Assessment of risk of bias

The quality of the studies that met eligibility criteria was independently assessed by two reviewers (CMV and UM). Ratings were then reviewed to discuss discrepancies. Quality assessment was conducted using the Effective Public Health Practice Project Quality Assessment Tool (EPHPP). This is a generic tool used to evaluate a variety of intervention study designs such as randomized controlled trials (RCTs) and before-and-after studies. This tool has been considered suitable to be used in systematic reviews of effectiveness [42]. The tool assesses six domains: (i) selection bias, (ii) study design, (iii) confounders, (iv) blinding, (v) data collection method, and (vi) withdrawals/dropouts. The tool guidelines indicate that each domain can be rated as strong, moderate, or weak. Based on the total score studies can be assigned a quality rating of strong, moderate, or weak.

## Results

A total of 7286 studies were identified after duplicates were removed. Once titles and abstracts were screened, 181 studies were selected for full text screening (Fig. 1). Finally, a total of eight studies met inclusion criteria and were included in the review [43–50].

### Study characteristics

A complete description of study characteristics is provided in Table 1. Of the eight included studies, six were conducted in the United States [43–45, 47–49], one in the United Kingdom [46], and one in Spain [50]. Six were RCTs [43, 45–47], and two were before-and-after studies [44, 48]. Regarding the type of substance assessed, three targeted smoking behaviour [43, 47, 50]; two targeted opiate dependence [44, 45]; two focused on alcohol or drug dependence [46, 49]; and finally, one on crystal methamphetamine abuse [48]. Six studies provided biochemical verification of substance use [43–45, 47, 49, 50]. The assessment points ranged from baseline to 12 months post-intervention.

**Table 1:** Characteristics of studies included

Author year	Study type	Setting and Country	Substance	Aim	Inclusion criteria	Sample characteristics
Daughters et al. (2018) [49]	RCT	Substance use residential treatment setting, USA	Alcohol/other drugs	To compare outcomes for a BA group treatment for substance use (LETS ACT) versus a time and group size-matched control condition delivered in a residential treatment setting.	Patients at the residential substance use treatment facility: (1) court-mandated to attend the program by the criminal justice system or (2) entered treatment voluntarily and received public funding.	*N* = 263 (female 29.5%, mean age 42.7; *SD* = 11.8) African American 95.4%
Gonzalez-Roz et al. (2018) [50]	RCT	Clinical Unit of Addictive Behaviours of the University of Oviedo, Spain	Tobacco	To analyze if adding a CM component to CBT and BA would increase smoking cessation treatment adherence and decrease depressive symptoms	(1) Age ≥ 18; (2) smoking ≥10 cigarettes per day within the last year; (3) meeting criteria for current unipolar major depression disorder (meeting DSM-IV-TR criteria or BDI score ≥ 14); and (4) meeting DSM-IV-TR criteria for nicotine dependence.	*N* = 74 (females 82.4%)
Busch et al. (2017) [43]	RCT	Inpatient cardiac units at The Miriam and Rhode Island Hospitals. Providence, RI, USA	Tobacco	To compare BAT-CS to a Standard-of-Care control on smoking abstinence, mood, and stress related variables.	(1) ACS diagnosis documented in medical record; (2) smoking ≥3 cigarettes/day prior to hospitalization; (3) age 18–75; (4) English fluency; (5) telephone access; (6) living within 1-h drive of admitting hospital; and (7) willingness to attempt to quit smoking at discharge.	*N* = 59 (female 27.1%; mean age 55.6; *SD* = 10.2) Non-Hispanic Caucasian 89.8%; Non-Hispanic African American 5.1%; Hispanic Caucasian 3.4%; Multiracial 1.7%
Delgadillo et al. (2015) [46]	RCT	CDAT services Leeds, UK	Alcohol/other drugs	To examine the feasibility of a 12-session face-to-face BA intervention compared to a CBT-based guided self-help intervention for depression.	(1) ≥1 month registered with CDAT; (2) clinically significant depression symptoms as defined by the PHQ-9; (3) mild-to-moderate symptoms of alcohol/drug dependence as defined by SDS.	*N* = 50 (female 32.0%; mean age 37.2; *SD* = 6.6) White British 72.0%, other 28.0%
Mimiaga et al. (2012) [48]	Before-and-after	The Fenway Institute, Fenway Health. Boston, MA, USA	Crystal methamphetamine	To evaluate BA with integrated HIV RR counseling for crystal methamphetamine abuse	(1) Age ≥ 18; (2) self-reported ≥1 episodes of unprotected anal sex with a nonmonogamous male sexual partner with concurrent use of crystal methamphetamine in the past 3 months; (3) HIV uninfected.	*N* = 19 (male 100%; mean age 40.0; *SD* = 9.5) Caucasian 62.5%; racial/ethnic minorities 37.5%
MacPherson et al. (2010) [47]	RCT	Not reported	Tobacco	To examine BA as a treatment for smoking cessation and depression vs. ST.	(1) Age18–65; (2) current regular smoker (≥1 year); (3) smoking ≥10 cigarettes/day; (4) BDI-II ≥10; (5) no current DSM-IV disorder assessed by the SCID-NP.	*N* = 68 (female 48.5%) BATS: mean age 45.0 (*SD* = 12.20); African-American 69.7% ST: mean age 42.6 (*SD* = 11.5); African-American 75.8%
Carpenter et al. (2008) [45]	RCT	Community-based treatment programs. New York City, NY, USA	Opiate	To test the efficacy of BTDD vs. REL for DSM-IV depressive disorders and substance abuse.	(1) Current DSM-IV major depression or dysthymic disorder; (2) stable methadone dose (no changes in prior two weeks) of ≥60 mg.	*N* = 38 (female 42.1%) BTDD: mean age 38.8 (*SD* = 10.40; Caucasian 88.9% REL: mean age 41.2 (*SD* = 10.9); Caucasian 75.0%
Carpenter et al. (2006) [44]	Before-and-after	2 community-based methadone maintenance programs. New York City, NY, USA	Opiate	To develop and pilot test a behavioural therapy for depression in drug dependence.	DSM-IV criteria for major depression or dysthymic disorders that: (1) antedated the earliest lifetime substance abuse; (2) persisted during 6 months of abstinence in the past, or at least 1 month during methadone treatment; (3) had a stable methadone dose of ≥60 mg; and (4) consented to participate.	*N* = 29 (female 41.3%; mean age 41.0; *SD* = 7.9) Caucasian 58.6%; Hispanic 27.6%; African-American 10.3%; and Asian 3.4%

*ACS* Acute Coronary Syndrome, *BA* Behavioural Activation, *BAT-CS* Behavioural Activation Treatment for Cardiac Smokers, *BDI-II* Beck Depression Inventory-II, *BTDD* Behavioural Therapy for Depression in Drug Dependence, *CBT* Cognitive-Behavioural Treatment, *CDAT* Community Drugs and Alcohol Treatment, *CM* Contingence Management, *DSM-IV-TR* Diagnostic and Statistical Manual of Mental Disorders, *LETS ACT* Life Enhancement Treatment for Substance Use, *MDD* Major Depression Disorder, *PHQ-9* Patient Health Questionnaire, *RCT* Randomised Controlled Trial, *REL* Structured Relaxation Intervention, *RR* Risk Reduction, *SCID-NP* Structured Clinical Interview for DSM-IV, non-patient version *SDS* Severity of Dependence Scale, *ST* Standard Treatment

### Methodological quality assessment

Overall, one study received a methodological quality rating of strong [49], six studies of moderate [43, 45–48, 50], and one study of weak [44]. The quality assessment ratings for each specific criterion and the assigned global rating are reported in Table 2. Study design and data collection dimensions were the main strengths of included studies, while blinding was the main weakness. Only in two studies [47, 49] participants and research staff assessing outcomes were blind to the study conditions. However, it is of note that blinding participants in behavioural intervention studies is often not feasible, as a result of the nature of the intervention.

**Table 2:** Ratings of methodological quality by EPHPP tool

	Selection bias	Study design	Confounders	Blinding	Data collection	Withdrawals	Global rating
Daughters et al. (2018) [49]	Strong	Strong	Strong	Strong	Strong	Strong	Strong
Gonzalez-Roz et al. (2018) [50]	Strong	Strong	Strong	Weak	Strong	Strong	Moderate
Busch et al. (2017) [43]	Strong	Strong	Strong	Weak	Strong	Strong	Moderate
Delgadillo et al. (2015) [46]	Moderate	Strong	Strong	Moderate	Strong	Weak	Moderate
Mimiaga et al. (2012) [48]	Strong	Moderate	Weak	Moderate	Strong	Strong	Moderate
MacPherson et al. (2010) [47]	Moderate	Strong	Strong	Strong	Strong	Weak	Moderate
Carpenter et al. (2008) [45]	Moderate	Strong	Moderate	Moderate	Strong	Moderate	Moderate
Carpenter et al. (2006) [44]	Weak	Moderate	Weak	Weak	Strong	Moderate	Weak

The EPHPP tool provides two additional methodological dimensions (intervention integrity and analyses), which were also considered. Only three studies provided information about the intervention integrity by assessing the percentage of participants who received the intervention as intended: two were scored in the 80–100% category [43, 50]; and one was scored in the less than 60% category [46]. With regard to the analysis component, all studies used intent-to-treat analyses as appropriate, except for Mimiaga et al. [48] who did not provide this information.

### Effects of BA intervention in substance use and depression outcomes

Intervention descriptions and a summary of the main findings of the effects of BA on substance use and depression outcomes are reported in Table 3.

**Table 3:** Intervention descriptions and main outcomes

Author (year)	BA intervention description	Control condition description	Therapist Session number (duration)	Depression outcomes	Substance use outcomes	Conclusion
Daughters et al. (2018) [49]	LET’S ACT: (1) to generate, schedule, engage in and record value-driven substance-free behaviours that serve to increase daily positive reinforcement; (2) to identify important life areas, values and activities that aid in the movement from a maladaptive response to negative mood to an increase behaviours that facilitate positive reinforcement.	Supportive counselling: therapist provided unconditional support, utilized reflective listening techniques and managed group dynamics by encouraging equal participation among patients. Participants established a list of continually evolving discussion topics.	Clinical psychology doctoral students and post-doctoral fellows trained in both conditions. Session length: five or eight sessions (60 min each session), balanced across conditions.	No significant changes in depressive symptoms by condition, time or their interaction. Significant time x abstinence interaction. Participants who were abstinent from pre-treatment to 12-month follow-up reported significantly fewer depressive symptoms at 12-months compared to substance users.	Abstinence rates were significantly higher for LETS ACT compared to the control condition at 3, 6 and 12 months follow-up.	LET’S ACT is an effective intervention to reduce the incidence of post-treatment substance use and substance use-related adverse consequences.
Gonzalez-Roz et al. (2018) [50]	CBT + BA: (1) BA treatment rationale; (2) psycho-education about the association between smoking and depression; (3) identification of life areas for generating meaningful, reinforcing and positive activities; and (4) encouraging to engage in and monitor each planned in-session activity.	CBT-BA + CM: Included components of CBT + BA and also reinforcing abstinence through earn points exchangeable for rewards on a schedule of escalating magnitude of reinforcement.	Master- and doctoral-level psychologists with experience in smoking cessation treatments, and trained in the specific treatments used in the study. Session length: eight weekly sessions (90 min each session).	There was a significant reduction in depressive symptoms from pre- to post-treatment. No significant differences between conditions were found in depression symptoms.	No significant differences were found between conditions in abstinence rates.	Adding a CM protocol to CBT-BA resulted in better treatment retention although it did not improve abstinence rates.
Busch et al. (2017) [43]	BAT-CS: (1) increasing pleasant and/or meaningful activities; (2) increasing activities for a non-smoking lifestyle; and (3) developing specific steps for a quit attempt.	SC: five mailings of 10 smoking cessation educational brochures.	Licensed clinical psychologist and clinical psychology post-doctoral fellow Session length: All participants: one smoking cessation session at the hospital (50 min). BAT-CS: a minimum of five post-discharge contacts at 1, 3, 6, 9, and 12 weeks (duration not specified).	No significant differences were found in depression from baseline to end-of-treatment, or at 24-week follow-up.	AOR favoured BAT-CS at the end-of-treatment and at 24-week follow-up. Mean number of days to first lapse and to first relapse after discharge was significantly greater for BAT-CS.	Preliminary evidence favouring BA and standard smoking cessation counselling combination for depressed mood and smoking cessation in patients following ACS.
Delgadillo et al. (2015) [46]	BA: (1) self-monitoring of depressive and maladaptive behaviours; (2) activity scheduling to increase and reinforce adaptive behaviour patterns; (3) reducing avoidant behaviours, rumination and maladaptive coping strategies.	GSH: to describe and encourage participants to apply a self-help booklet for depression based on CBT principles.	Qualified psychological well-being practitioners trained in BA (postgraduate level in structured guided self-help interventions, 1 year supervised clinical training course) and CDAT workers who delivered GSH (trained by a counseling psychologist). Session length: BA: 12 sessions (duration not specified). GSH: one session (60 min).	Moderate and comparable improvements in depressive symptoms over time were found for participants in both treatment groups.	There was a reduction in substance use in the BA group, but the difference was not statistically significant.	Psychological interventions integrated within CADT are needed to improve patients’ mental health.
Mimiaga et al. (2012) [48]	BA-RR: (1) building rapport, treatment rationale, and gathering information about participant’s patterns of substance use, mental health history, and substance use treatment history; (2) information and motivation to sexual risk reduction; (3) BA integrated with risk-reduction counselling; (4) review and relapse prevention planning.	No comparison group.	Therapist level not reported. Session length: 10 sessions (50 min each session).	Significant reductions in depression scores from baseline to acute post-intervention and to 3-month follow-up.	Significant reductions in crystal methamphetamine use and polysubstance use.	An integrated behavioural program may impact sexual risk, substance use, and depression outcomes.
MacPherson et al. (2010) [47]	BATS: (1) structuring reinforcing activities; (2) activity monitoring; (3) identification of values and life goals; (4) planning activities; (5) recording the engagement in planned activities; (6) activities related with smoking cessation process and to stay abstinent, addressing lapses, and coping with triggers; (7) incorporating non-smoking lifestyle activities.	ST: self-monitoring, identifying cessation strategies from prior quit attempts, relaxation, coping with triggers, identifying social support for cessation, making lifestyle changes, and homework.	Clinical psychologist (doctoral degrees and clinical psychology doctoral students), trained for both conditions. Session length: 8 sessions (30 min of BA and 30 min of core ST components each session).	A reduction in depressive symptoms from baseline to 26-week post assigned quit date was observed. The reduction in depressive symptoms over time was greater for BATS than for ST participants.	BATS showed greater odds of smoking abstinence during the follow-up period compared to ST.	BATS is a promising intervention for smoking cessation and reduction of depression among smokers with depressive symptoms.
Carpenter et al. (2008) [45]	BTDD: (1) increasing the frequency and/or breadth of pleasant activities; (2) assessment of the relation between mood and pleasant activities; (3) rating frequency and pleasure of activities, and satisfaction in 9 life areas; (4) weekly definition of out-of-session activities to increase the amount of pleasant activities.	REL: (1) progressive muscle relaxation, (2) autogenic relaxation exercises and, (3) visual imagery.	Trained therapist. Session length: 24 weekly sessions (duration not specified) for both conditions.	Depression decreased during treatment. The average depression ratings at end of treatment were equivalent across treatments.	In both treatment conditions there was a significant increase in the odds of benzodiazepine use, and a significant decrease in the odds of opiate use.	REL and BTDD targeting depressive and substance use disorders facilitate clinical improvement.
Carpenter et al. (2006) [44]	BTDD: (1) education about the relation between mood and activity level; (2) increasing activities in relevant life areas; (3) developing skills to increase activities; and (4) CM for therapy adherence and completion of therapeutic activities.	No comparison group.	Trained therapist. Session length: 16 individual sessions (duration not specified) over 24-weeks.	Significant decrease in self-rated and clinician-rated depression at weeks 12 and 24. During treatment 48.30% of patients demonstrated ≥50% reduction in HAMD.	There were no significant changes in opiate and cocaine use. Treatment responders reported a significant reduction on BZ use.	A behaviourally based treatment for depression seeking to increase rewarding activities in targeted life areas is associated with a significant reduction in depression severity.

*ACS* Acute Coronary Syndrome, *BA-RR* Behavioural Activation Therapy and Risk Reduction Counselling Intervention, *BAT-CS* Behavioural Activation Treatment for Cardiac Smokers, *BATS* Behavioural Activation Treatment for Smoking, *BTDD* Behavioural Therapy for Depression in Drug Dependence, *BZ* Benzodiazepine, *CDAT* Community Drugs and Alcohol Treatment, *CM* Contingence Management, *GSH* Guided Self-help, *HAMD* Hamilton Depression Scale, *LETS ACT* Life Enhancement Treatment for Substance Use, *PDA* Percentage of Days Abstinent, *REL* Relaxation, *SC* Standard Care, *ST* Standard Treatment

### Substance use outcomes

Two of the six RCT included found significantly higher abstinence rates for BA compared to the control condition in each point assessment [47, 49]. Specifically, Daughters et al. [49] reported abstinence ORs of 2.2, 2.6, and 2.9 at 3, 6, and 12 months respectively in the BA condition. MacPherson et al. [47] also found significant OR in the BA condition (OR = 4.0 at 1 week post-quit; 2.06 at 4 weeks; 2.71 at 16 weeks, and 3.59 at 26 weeks).

No significant differences in abstinence rates were found between BA and the control conditions in the rest of the RCT included [43, 45, 46, 50]. However, in one study mean number of days to first lapse after discharge was significantly higher for BA when comparing to the control condition (62.4 vs. 31.8 days, respectively, *p* = .03) [43]. Lastly, the study conducted by Delgadillo et al. [46], found 17% increase of days abstinent after treatment in the BA group. This indicates that there was a reduction in substance use in the BA group, whereas no change was detected in the control group [46]. Although there was a positive trend associated with the BA condition, differences were not statistically significant (Mean differences between-group effect size of *d* = 1.52, *p* = .08).

Regarding the two studies that compared pre- and post- substance use rates the results were mixed. While Carpenter et al. [44] did not find changes in opiate and cocaine use after treatment, Mimiaga et al. [48] found a significant decrease from baseline to acute post-intervention and to 3 months post-intervention in the number of days of use in the past 30 days (*p =* .010), in number of crystal methamphetamine episodes in the past 3 months (*p <* .001), and in number of days experiencing drug-related problems during the past 30 days (*p = .*005).

### Depression outcomes

The majority of studies included in the present review found a significant improvement in depression symptoms over time [44–48, 50]. However, most studies showed equivalent results across treatment conditions [45, 46, 49, 50]. Only one RCT [47] found a significant reduction in depression symptoms for those participants randomized to BA compared to the control group (*B* = − 1.99, *SE* = 0.86, *p =* .02). Moreover, depressive symptoms declined significantly from baseline to the 26-week post assigned quit-date (*B* = − 1.53, *SE* = 0.68, *p =* .03).

Interestingly, one RCT study found a reduction in depression over time but only among abstainers regardless of the treatment condition [49]. Participants who remained abstinent at 12-month follow-up reported significantly fewer depressive symptoms, compared to substance users (*B* = − 5.74, SE = 1.65, 95% CI = − 9.10, − 2.58). In addition, they found a significant decrease in depressive symptoms from pre-treatment to 12-months post-treatment only in abstainers (*B* = − 0.43, SE = 0.11, 95% CI = − 0.65, − 0.22).

## Discussion

The aim of this systematic review was to examine if BA has an effect in reducing substance use and depression. Previous research has shown that reinforcement processes play a central role in the onset, maintenance, and recovery from depression [38] and SUDs [18]. Both disorders share features such as a reduced engagement in enjoyable non-drug-related activities/reinforcement or the presence of anhedonia, defined as a diminished interest/pleasure in response to previously rewarding activities [23, 51]. Since the main focus of BA is to increase healthy and rewarding activities [37], the potential use of BA in substance use treatment is justified.

Overall, the results of the present review were mixed. Although some studies indicated that BA reduced significantly substance use [47, 49] and depression [47], the effect sizes were moderate [43, 46]. In addition, most of studies included did not reach statistical significance [43, 46, 50]. Since the majority of the studies were pilot [43–45, 47] or feasibility studies [46], they may be underpowered to detect significant differences. In fact, the only well powered RCT [49] showed that BA significantly reduced substance use.

Interestingly, BA has demonstrated its effectiveness for depression treatment [34]; although for people with substance use and depression, BA effect seems to be larger for substance use than for depression when compared against a control condition. It is possible that substance use outcomes (e.g., abstinence status) after treatment or during the follow-up period had an impact in depression outcomes, since previous research has found an association between substance use abstinence and depressive symptoms reduction [52–54]. Given that the majority of studies have analyzed substance use and depression outcomes separately, it would be interesting to analyze their interaction in future studies. Moreover, as suggested by Daughters et al. [49], research is needed to examine the effect of BA-based interventions on primary versus secondary depression in people with comorbid substance use and depression.

In addition, diverse factors could have influenced in the results found. One of them could be the heterogeneity of inclusion criteria for the different studies. For example, Busch et al. [43] and Mimiaga et al. [48] included participants with a wide range of baseline depression scores, from asymptomatic to individuals with severe symptomatology; whereas Carpenter et al. [44] and Carpenter et al. [45] only included participants with a DSM-IV diagnosis of major depression or dysthymic disorder. Measures for depression were also heterogeneous (e.g., Beck Depression Inventory-II; Hamilton Depression Scale; Montgomery–Åsberg Depression Rating Scale; Patient Health Questionnaire), as well as the target population (e.g., Acute Coronary Syndrome [ACS] patients, Human Immunodeficiency Virus [HIV] uninfected men who have sex with men), the treatment setting (e.g., inpatient cardiac units, community-based methadone maintenance programs; community drugs and alcohol treatment services), or the different stages of drug use treatment. Control conditions, type, length, and intensity also vary significantly across studies. Carpenter et al. [45] used a structured psychological treatment as a comparison group (e.g., 24 face-to-face weekly sessions of relaxation intervention), whereas Busch et al. [43] used a Standard-of-Care condition (e.g., one face-to-face session and five emails of printed educational material about smoking cessation). Baseline significant differences between groups could also have influenced the results. In fact, in the study conducted by Carpenter et al. [45], the Behavioural Therapy for Depression in Drug Dependence (BTDD) condition had a greater proportion of opiate users (*p* ≤ .03), and in the study conducted by Delgadillo et al. [46] baseline Severity of Dependence Scale was also significantly higher in the BA group (*p* = .03). Finally, the studies included in this review used different BA treatment approaches. Concretely, we found that two studies [44, 45] used an approach based on Lewinsohn et al. conceptualization [55], two studies [46, 48] used the Martell et al., BA approach [56], whereas the remaining four studies [43, 47, 49, 50] used a modified version of the brief behavioural activation treatment for depression (BATD) [39]. All these approaches are based on the principles of the behavioural model, share the use of behavioural strategies, and focus on behaviour change [40], but they have some differences. For example, the BA conceptualization of Lewinsohn et al. [55] focuses on assessing the relationship between mood and pleasant activity level, and on increasing the frequency of pleasant activities to facilitate positive interactions between the individual and the environment. The BA protocol of Martell et al. [38] focuses on behaviour functional analysis (examining antecedents and consequences) in order to identify behavioural avoidance patterns (e.g., avoid trying new activities or avoid attending social events/activities), and includes the use of strategies as mental rehearsal, periodic distraction or skill-training. In the case of the BATD model of Lejuez et al. [39], it focuses on increasing reinforcement for non-depressive behaviours (e.g., sport-related activities, social or leisure activities) emphasizing the personal value of these alternative behaviours. Further research is needed to determine if different BA treatment approaches have the same effects on depression and substance use outcomes.

Other limitations of this review include: first, the length of participants’ follow-ups. Only one study included a 12 months follow-up [49], while the rest had the longest follow-up at 24 weeks post-intervention, which limited examining the long-term sustainability of treatment effects. Second, since BA effectiveness has been widely demonstrated in depression treatment [34, 57], we sought to investigate whether BA could improve not only depression, but also substance use outcomes. Thus, only studies that provided both outcomes were included. For this reason, we excluded two studies examining the effects of a BA intervention, named LETS Act!, on depression in standard inpatient substance abuse treatment [58, 59]. Although they were excluded, the results of both studies suggest that the BA approach reduce depressive symptoms in this specific population, and one of them [59] also found a significantly lower percentage of individuals that dropped out of residential substance abuse treatment in the LETS Act! condition. These findings provide additional support to the positive effects of the BA approach.

Regarding the quality of the studies, it is of note that only one of the studies reached the qualification of strong methodological quality [49]. Further high-quality studies are needed to improve confidence in these findings and to confirm the positive effect of BA both in substance use outcomes and depression. In line with our results, a recent overview about cognitive-behavioural therapies for substance use and depression disorders conducted by Vujanovic et al. [28] showed that, despite the growing evidence supporting the effectiveness of integrated CBT for the treatment of co-occurring SUD-depression, the scarce of well-controlled studies limit their conclusions. Finally, six of the eight studies were conducted in the United States, which should be considered in the interpretation and generalization of results, as it has not been tested in others geographical and cultural settings.

Despite the limitations, this systematic review clearly described and followed internationally accepted standards for the process of identifying studies. In addition, despite that BA has demonstrated its effectiveness in the treatment of depression treatment, this review addresses its comorbidity with substance use. This is a novel and relevant topic since depression influences SUDs recovery and relapse. In addition, SUDs imply in many cases a lack of natural and alternative reinforcers and activities that are meaningful in life and provide a sense of purpose [35], which can have an impact in substance use-related behaviour change.

Although more research is needed to support the effectiveness of BA for the treatment of substance use and depression, the studies reviewed showed promising and suggestive data. Future studies are required to investigate the mechanisms of action of BA, as well as possible moderator variables that can have impact in SUDs and depression outcomes. More research is also needed to investigate whether the BA model can just be applied to SUDs and whether the reward-related processes in SUDs and depression are as comparable as implied. For example, it is necessary to elucidate whether there are differences between anticipatory anhedonia (e.g., diminished subjective desire, interest, and anticipation of pleasant stimulus/activity) and consummatory anhedonia (e.g., inability to experience pleasure in response to a pleasurable stimulus/activity) in SUDs and depression, and if this could influence treatment outcomes. Finally, future studies should be conducted on cost-effectiveness of this intervention approach, and on how BA can be implemented into clinical and community settings.

## Conclusions

The results of this systematic review suggest that BA may help to improve substance use and depressive symptoms. However, research into BA in substance use and depression is at an early stage, and the majority of results are based on pilot studies with methodological limitations. Thus, they should be interpreted with caution. Given the high comorbidity of substance use and depressive symptoms, and the preliminary results indicating that BA may be a useful intervention for this population, more research is required to establish BA effectiveness. Future studies should be conducted adhering to standard reporting guidelines and using rigorous methodology including sample size calculations, adequate methods of randomization, intention-to-treat analysis, and longer follow-up periods.

In summary, BA is a promising option that could be easily integrated in substance use treatments due to its brevity and parsimony. BA could be implemented in treatment and community programs that make accessible and provide the opportunity to participate and engage in social, healthy, and cultural activities, offering more options for substance-free sources of reinforcement. Compromise and economic resources of governments and policymakers’ result essential to make possible to deal with substance use and depression, as these problems have an enormous cost at personal, social, and economic levels.

## Additional files

Additional file 1: PRISMA Checklist. (DOCX 29 kb)

Additional file 2: Literature search strategy. (PDF 460 kb)

## Abbreviations

ACS: Acute coronary syndrome
BA: Behavioural activation
BAT-CS: Behavioural activation treatment for cardiac smokers
BDI-II: Beck depression inventory-II
BTDD: Behavioural therapy for depression in drug dependence
CBT: Cognitive behavioural treatment
CDAT: Community drugs and alcohol treatment
EPHPP: Effective public health practice project quality assessment tool
MDD: Major depression disorder
PHQ-9: Patient health questionnaire
RCT: Randomized controlled trial
REL: Structured relaxation intervention
RR: Risk reduction
SCID-NP: Structured clinical interview for dsm-iv, non-patient version
SDS: Severity of dependence scale
ST: Standard treatment
SUDs: Substance use disorders
