# Correction to: Effects of behavioural activation on substance use and depression: a systematic review

**DOI:** 10.1186/s13011-020-00274-6

**Published:** 2020-05-31

**Authors:** Carmela Martínez-Vispo, Úrsula Martínez, Ana López-Durán, Elena Fernández del Río, Elisardo Becoña

**Affiliations:** 1 Smoking Cessation and Addictive Disorders Unit, Department of Clinical Psychology and Psychobiology, Faculty of Psychology, University of Santiago de Compostela, Santiago de Compostela, Spain; 2 Tobacco Research and Intervention Program, Department of Health Outcomes and Behaviour, H. Lee Moffitt Cancer Center, Tampa, FL USA; 3 Department of Psychology and Sociology, University of Zaragoza, Zaragoza, Spain

**Correction to: Subst Abuse Treat Prev Policy (2018) 13:36**

**https://doi.org/10.1186/s13011-018-0173-2**

Following publication of the original article [1], we have been notified that there are some parts in the article text that need to be changed as per below:

- Table 2 Ratings of methodological quality by EPHPP should change Blinding rating of Busch et al. (2017) [43] from Weak to Moderate and Global rating from Moderate to Strong;
- Abstract text part should be changed to: One trial received weak methodological quality, five moderate, and two strong;
- Results text part in Methodological quality assessment should be changed to: Overall, two studies received a methodological quality rating of strong [43, 49], five studies of moderate [45–48, 50], and one study of weak [44];
- Discussion text part should be changed to: Regarding the quality of the studies, it is of note that only two of the studies reached the qualification of strong methodological quality [43, 49].
