# Supplementary material for: Effects of behavioural activation on substance use and depression: a systematic review
Source: Subst Abuse Treat Prev Policy. 2018 Sep 29;13:36. doi: 10.1186/s13011-018-0173-2 (PMC6162964; doi:10.1186/s13011-018-0173-2)
Supplement: Supplementary file 2 — Literature search strategy. (PDF 460 kb) [file 13011_2018_173_MOESM2_ESM.pdf]

	Searches MEDLINE
1	((drug* OR substance OR caffeine) AND (abus* OR misuse* OR addict* OR dependen* OR disorder* OR overdose OR intoxicat* OR abstin* OR abstain* OR withdrawal* OR bing*))
2	“Drug User*” OR “energy drink*”
3	“Substance-Related Disorder*”
4	“Amphetamine-Related Disorder*”
5	“Cocaine-related disorder*”
6	“Inhalant abus*”
7	“Marijuana Abus*”
8	“Opioid-related disorder*”
9	“Substance Abuse, Intravenous”
10	“Street drug*”
11	“Substance Withdrawal Syndrome*”
12	“Substance Abuse Treatment Center*”
13	“Heroin Dependen*”
14	1 OR 2 OR 3 OR 4 OR 5 OR 6 OR 7 OR 8 OR 9 OR 10 OR 11 OR 12 OR 13
15	“depression”
16	“depressive”
17	depress*
18	Mood
19	Affect*
20	dysthym*
21	antidepress*
22	15 OR 16 OR 17 OR 18 OR 19 OR 20 OR 21
23	“behav* activation”
24	“behav* treatment”
25	“behav* intervention”
26	“behav* therapy”
27	“activit* scheduling”
28	“pleasant activit*”
29	23 OR 24 OR 25 OR 26 OR 27 OR 28
30	14 AND 22 AND 29
	limit to humans

	Searches PSYCINFO
1	(drug* OR substance OR caffeine) N/2 (usage OR abus*OR addict* OR dependen* OR overdose OR intoxicat* OR abstin* OR abstain* OR withdrawal* OR bing*)
2	Drug* N/0 User*
3	street N/0 drug*
4	energy N/0 drink*
5	Substance* N/0 (abus* OR use*)
6	Amphetamine* N/0 (abus* OR use*)
7	Cocaine N/0 (abus* OR use*)
8	Inhalant N/0 (abus* OR use*)
9	Cannabis N/0 (abus* OR use*)
10	Opi* N/0 (abus* OR use*)
11	Intravenous N/0 drug* N/0 (abus* OR use*)
12	Drug* N/0 Withdrawal
13	Heroin N/0 addiction (abus* OR use*)
14	1 OR 2 OR 3 OR 4 OR 5 OR 6 OR 7 OR 8 OR 9 OR 10 OR 11 OR 12 OR 13
15	“Major depression”
16	depressive
17	depress*
18	Mood
19	Affect* N/0 disorder*
20	dysthym*
21	antidepress*
22	15 OR 16 OR 17 OR 18 OR 19 OR 20 OR 21
23	behav* N/0 activation
24	behav* N/0 treatment*
25	behav* N/0 intervention*
26	behav* N/0 therap*
27	Activit* N/0 scheduling
28	pleasant N/0 activit*
29	23 OR 24 OR 25 OR 26 OR 27 OR 28
30	12 AND 20 AND 27
	Limit to Human
	Limit so research journals (books and dissertations excluded)

# ▲	Searches EMBASE
1	((dru$ or substance$ or caffeine) and (abus$ or misuse$ or addict$ or dependen$ or disorder$ or overdose or intoxicat$ or abstin$ or abstain$ or withdrawal$ or bing$)).af.
2	("drug user$" or "energy drink$").af.
3	(substance adj related adj disorder$).af.
4	(cocaine adj related adj disorder$).af.
5	(amphetamine adj related adj disorder$).af.
6	(inhalant adj abuse$).af.
7	(marijuana adj abuse$).af.
8	(opi* adj related adj disorder$).af.
9	(substance adj abuse$ adj intravenous).af.
10	(street adj drug$).af.
11	(substance adj withdrawal adj syndrom$).af.
12	(substance adj abuse adj treatment adj center$).af.
13	(heroin adj dependen*).af.
14	1 or 2 or 3 or 4 or 5 or 6 or 7 or 8 or 9 or 10 or 11 or 12 or 13
15	(major adj depression).af.
16	depressive.af.
17	depress$.af.
18	mood.af.
19	(affect$ adj disorder$).af.
20	dysthym$.af.
21	antidepress$.af.
22	15 or 16 or 17 or 18 or 19 or 20 or 21
23	(behav$ adj activation).af.
24	(behav$ adj treatment$).af.
25	(behav$ adj intervention$).af.
26	(behav$ adj therap$).af.
27	(activit$ adj scheduling).af.
28	(pleasant adj activit$).af.
29	23 or 24 or 25 or 26 or 27 or 28
30	14 and 22 and 29
	limit 30 to human
	limit to exclude medline journals
